# Editorial: Unveiling microbiome interactions and functions in soil hotspots

**DOI:** 10.3389/fmicb.2026.1820854

**Published:** 2026-05-20

**Authors:** Agnieszka Kuźniar, Alok Prasad Das, Weronika Goraj

**Affiliations:** 1 Laboratory of Genomics and Genetics, Faculty of Medicine, The John Paul II Catholic University of Lublin, Lublin, Poland; 2 Department of Life Sciences, Rama Devi Women's University, Bhubaneswar, India; 3 Department of Microbiology and Translational Medicine, Faculty of Medicine, The John Paul II Catholic University of Lublin, Lublin, Poland

**Keywords:** hotspot, metagenomics, microbiome, microorganism, soil, ecological niche

Soil hotspots represent microscale environments characterized by elevated biological activity, intensified biogeochemical cycling, and highly dynamic microbial interactions. Despite their limited spatial extent, these localized zones exert a disproportionate influence on ecosystem functioning, regulating nutrient turnover, greenhouse gas emissions, plant productivity, and soil resilience. Found in rhizospheres, detritus rich microsites, soil aggregates, and biogeochemical interfaces, hotspots act as focal points where microbial diversity, metabolic activity, and ecological interactions converge.

The Research Topic “*Unveiling Microbiome Interactions and Functions in Soil Hotspots*” brings together 21 manuscripts that collectively advance our understanding of soil microbiomes across environmental gradients, land use systems, and ecological contexts. These contributions span agricultural, forest, degraded, and extreme environments, providing a comprehensive perspective on how microbial communities respond to both natural and anthropogenic drivers. Several studies highlight the importance of abiotic factors and temporal variability in shaping microbial communities. Casagrande Pierantoni et al. demonstrate that seasonal variation exerts a stronger influence than crop identity on microbial community structure in rotational systems. This finding emphasizes temporal heterogeneity as a defining characteristic of hotspot activity. Similarly, Wang et al. show that forest harvesting strategies significantly influence microbial diversity in *Larix gmelinii* forests, revealing the sensitivity of soil microbiomes to management practices. Jiang et al. further demonstrate that intercropping modifies rhizosphere microbial communities, although these effects remain strongly mediated by environmental conditions. Together, these studies indicate that while management practices shape microbial assemblages, environmental drivers such as temperature, soil chemistry, and moisture often play a dominant role. Soil hotspots therefore represent dynamic systems, continuously responding to fluctuating abiotic conditions. Nitrogen cycling is a central process within soil hotspots. Sun et al. show that AM fungi selectively suppress ammonia oxidizing bacteria and *Nitrospira* sp., while having limited impact on ammonia oxidizing archaea. This selective regulation suggests that mycorrhizal associations can reshape nitrification pathways and influence nitrogen retention. Complementing this, Li et al. demonstrate that continuous monocropping leads to microbial degradation and functional decline, highlighting the vulnerability of nitrogen transforming communities under sustained agricultural pressure. These findings collectively underscore that nitrogen cycling within hotspots is tightly regulated by both biological interactions and management practices, with important implications for nutrient-use efficiency and environmental sustainability. Plant–microbe interactions emerge as a unifying theme across the Research Topic. The rhizosphere serves as a key hotspot environment where plants actively shape microbial communities. Jiang et al. show enhanced microbial diversity and functional potential in intercropped systems. Ibrahim et al. further demonstrate that plant spatial arrangement influences AMF diversity and community structure. Cui et al. highlight the role of salt tolerant vegetation in restructuring microbial networks and improving soil conditions. Hou et al. link microbial communities to plant secondary metabolite production, illustrating functional feedbacks between plants and microbiomes. Díaz-Santiago et al. identify a conserved core microbiome, suggesting functional redundancy across environments. Magagula et al. demonstrate how disease alters rhizosphere microbial structure, while Sakha et al. characterize AMF diversity in stress-prone ecosystems. Together, these studies emphasize that plants actively engineer microbial hotspots, influencing nutrient cycling, stress tolerance, and ecosystem resilience. A second major theme involves the manipulation of soil hotspots through targeted amendments. Li et al. demonstrate that organic amendments can reshape microbial communities and enhance crop quality. Jiang et al. show that waste-derived materials can support biological control. Craft et al. reveal that biopolymers improve soil structure and microbial activity. Jia et al. demonstrate that soil fauna enhance microbial stability and support restoration processes. These studies collectively highlight the potential of microbiome informed interventions to engineer beneficial hotspots and improve agricultural sustainability. Land use and long-term management significantly influence microbial community structure. Demyanyuk et al. report increased fungal diversity and pathogenicity under long-term cultivation. Wang et al. and Jia et al. further demonstrate that management and disturbance reshape microbial communities and their stability. Cui et al. highlight how microbial networks contribute to ecosystem recovery. Across these systems, increased connectivity and functional redundancy are associated with resilience, whereas disturbance often leads to simplified networks. Vasanthrao et al. identify polluted soils as anthropogenic hotspots characterized by altered microbial communities and increased antibiotic resistance genes. Lin et al. further demonstrate how chemical exposure reshapes fungal communities, emphasizing the sensitivity of soil microbiomes to anthropogenic inputs. Rozmiarek et al. show that microbial activity in permafrost strongly regulates methane emissions, highlighting the role of hotspots in climate feedbacks. Long et al. demonstrate that vegetation type influences microbial carbon fixation, linking plant cover to carbon cycling. Muzami et al. provide insights into microbial functional traits in tropical systems, emphasizing the role of microbiomes in sustaining agricultural productivity. A defining strength of this Research Topic lies in its integrative approach. By combining metagenomics, ecological theory, and network analysis, the included studies demonstrate how micro-scale microbial interactions scale up to ecosystem level processes.

Future research should focus on linking metagenomic potential to actual metabolic activity, refining network-based indicators of resilience, and expanding studies in climate-sensitive systems. Additionally, microbiome-informed management strategies hold promise for enhancing soil health and sustainability.

## Conclusions

Soil microbial hotspots, though microscale in dimension, exert macroscopic impacts on ecosystem functioning. The 21 studies included in this Research Topic collectively demonstrate that hotspot dynamics are governed by complex interactions among environmental drivers, biological processes, and anthropogenic pressures. As global change accelerates, understanding and managing these critical microenvironments will be essential for ensuring soil sustainability, food security, and climate stability.

